# Using CFIR framework for understanding barriers and facilitators to implementation of community tuberculosis program in Burkina Faso

**DOI:** 10.3389/frhs.2023.1294176

**Published:** 2024-01-05

**Authors:** Flore M. Gisèle Donessouné, Olivier G. Sossa, Seni Kouanda

**Affiliations:** ^1^ Department of Doctoral School of Public Health, University Joseph Kizerbo, Ouagadougou, Burkina Faso; ^2^ University Thomas Sankara, Ouagadougou, Burkina Faso; ^3^ Department of Public Health, Research Institute for Health Sciences (IRSS), Ouagadougou, Burkina Faso

**Keywords:** barriers, facilitators, implementation, community program, tuberculosis

## Abstract

**Introduction:**

In 2020, there were nearly 9.9 million new Tuberculosis cases and 1.3 million deaths, with about 95% occurring in developing nations. Burkina Faso implemented a community Tuberculosis program, involving Civil Society Organizations, to increase screening and improve treatment outcomes. Therefore, this study aims to identify the factors influencing the implementation of community interventions involving these organizations in the fight against TB in Burkina Faso.

**Method:**

This qualitative study conducted semi-structured key informant interviews with a purposive sample of health providers from the ministry of health and community health workers. We used framework (the consolidated framework for implementation research was used method to identify barriers and facilitators to implementation of community tuberculosis program in Burkina Faso.

**Results:**

A total of 23 interviews were conducted. The results of this research shed light on several key factors that either contributed to or hindered the program's success. Among the facilitating factors, we identified close collaboration between national and international stakeholders, as well as remarkable program flexibility to adapt to local conditions. Furthermore, continuous training and support for community health workers proved crucial for the program's implementation. However, significant challenges were also unveiled. These challenges encompassed insufficient financial resources, difficulties related to the recruitment and management of civil society associations, and issues regarding program ownership at the peripheral level. Additionally, irregular payments to community health workers had a detrimental impact on their motivation and commitment.

**Conclusions:**

Our study conducted a comprehensive examination of the obstacles and facilitators encountered in the implementation of a community-based tuberculosis control program in Burkina Faso. The results of this research shed light on several key factors that either contributed to or hindered the success implementation of program. Measures should be taken to mobilize national resources, strengthen the capacities of associations, and promote local ownership of the program. Special attention should also be given to improving financial management and resolving issues related to the recruitment and compensation of community health workers. For such community-based tuberculosis programs to succeed in Burkina Faso and in similar context it is essential to address these obstacles and facilitators.

## 1 Introduction

Tuberculosis (TB) continues to be a global health challenge, with the World Health Organization (WHO) estimating that a quarter of the world's population is infected with Mycobacterium tuberculosis. In 2020 alone, there were approximately 9.9 million new cases of active TB and 1.3 million deaths attributed to the disease. A significant majority of these cases, about 95%, were reported in developing countries (1). TB is often linked to poverty, exposing affected individuals to economic distress, vulnerability, marginalization, stigma, and discrimination.

Low-income health systems, particularly in sub-Saharan Africa, bear a substantial burden of tuberculosis (1). Burkina Faso, for instance, despite making efforts to control the spread of this infectious, disease continues to face high tuberculosis prevalence rates, particularly in rural and disadvantaged communities. In 2019, Burkina Faso reported an estimated tuberculosis incidence rate of 46 cases per 100,000 inhabitants, with a mortality rate of 9.7 cases per 100,000 inhabitants among HIV-negative TB patients. Moreover, there was a 24% proportion of missing TB cases, and the treatment success rate for new and relapsed patients stood at 81.5% (2).

To address this challenge, community involvement in health promotion has emerged as an effective strategy to enhance health system performance (3). The World Health Organization (WHO)'s Stop TB strategy has recognized the importance of empowering communities to fight tuberculosis as a vital condition for achieving its objectives (4). Engaging community volunteers in the detection process is expected to lead to earlier diagnoses and improved prospects for a full recovery, while also reducing the costs of care.

Data from thirty-eight countries indicate that, on average, 27% of TB patients were notified through Community Health Workers (CHWs) referrals, resulting in an 87% treatment success rate for patients followed by CHWs (3). Building on this success, Burkina Faso implemented an innovative community-based TB program, funded by the Global Fund, to increase screening and enhance treatment success. This program enlisted the involvement of Civil Society Organizations (CSOs) and Non-Governmental Organizations (NGOs) to carry out awareness activities, screening of presumptive TB cases, and community-based Directly Observed Treatment, Short Course (DOTS). The ultimate goal was to alleviate the burden of tuberculosis and save lives (5–7).

However, limited research to date has examined the conditions that facilitate or jeopardize the successful implementation of these kind of program of Community Based Organizations (CSOs and NGOs) in West Africa and their role in the fight against tuberculosis. Therefore, the objective of this study was to identify the factors influencing the implementation of community interventions involving NGOs and CSOs in the fight against tuberculosis in Burkina Faso. Understanding these factors becomes critical when implementing programs under less controlled conditions, such as real-world contexts.

Furthermore, it is essential to synthesize evidence of what has worked and what has not worked in the provision of TB services by CHWs to benchmark and adapt or adopt similar strategies in comparable country settings. Recently, we evaluated the fidelity of the community-based tuberculosis control program as implemented by Civil Society Organizations (CSOs) in Burkina Faso. The results showed a relatively good fidelity of the program. Community-based programs interface with both health and community systems, requiring optimal integration at different levels. Given the dynamic context and the potential challenges that may arise during implementation at various levels, exploring and comprehending the facilitators and barriers affecting the implementation of this community-based program in Burkina Faso is highly relevant.

## 2 Study methods

### 2.1 Description of the community TB intervention in Burkina Faso

The community-based TB program in Burkina Faso was implemented by Community-Based Organizations (CBOs), which are civil society organizations (8, 9). A total of 22 associations operating in 5 districts were involved in the program. Two members from each association were trained to carry out the program's activities. The program included the following components:

#### 2.1.1 Prevention activities

These activities focused on raising awareness, providing information, education, and communication (IEC), and promoting behavior change communication (BCC) within the community. The aim was to increase community knowledge about tuberculosis and encourage behavior changes that could prevent its spread.

#### 2.1.2 Diagnosis activities

The program involved screening and contact tracing to identify individuals who might be symptomatic of tuberculosis. Through active case finding, the program systematically searched for potential TB cases in the community. Presumptive TB cases were then referred to diagnosis and treatment centers (DTCs) located in health centers.

#### 2.1.3 Treatment adherence support and home care

Community volunteers were responsible for providing support to individuals who tested positive for TB. They visited these individuals to supervise the use of anti-TB drugs and ensure treatment adherence. This component aimed to ensure that TB patients received appropriate care and support within their communities.

The individuals trained to carry out these community activities were referred to as community health workers, community volunteers, or animators. The implementation of the program was supervised by two sub-recipients (SRs): SR1—BURCASO and SR2—URCB. The Principal Recipient (PR) for the Global Fund (GF) funding was the Support Program for the Associative and Community (PAMAC), which served as the financial management unit and provided supervision to the SRs.

### 2.2 Conceptual framework

To gain a comprehensive understanding of the implementation process, we employed the Consolidated Framework for Implementation Research (CFIR) developed by Damschroder et al. (8). This framework is used to retrospectively identify the factors influencing the implementation of an intervention. Thus, we applied the CFIR to assess both barriers and facilitators to the implementation of the TB community program. The CFIR was specifically designed to guide the systematic assessment of multi-level implementation contexts, aiming to determine factors that might influence the implementation and effectiveness of interventions.

The CFIR is a “meta-theoretical” framework that integrates theories, models, and implementation frameworks from the health field. It is relevant to our case as it allows us to delve into the intricacies of our community intervention. This comprehensive framework takes into account various contextual factors across multiple levels that can impact the implementation and ultimate success of the intervention. It encompasses 37 constructs distributed within 5 domains: (1) intervention characteristics; (2) outer setting; (3) inner setting; (4) characteristics of individuals; and (5) process (8). However, researchers like Damschroder et al. and Kirk et al. have recommended its adaptation to specific contexts (8, 9).

Since the CFIR has primarily been tested with interventions implemented in health facilities such as clinics and primary healthcare centers, we deemed it necessary to add dimensions to tailor it to more or less well-structured community settings. As a result, we made adjustments by removing sub-constructs from some domains. For instance, we excluded the “trialability” sub-construct from the domain of program characteristics as this program was already being scaled, making pilot testing unnecessary. Additionally, we introduced two new sub-domains:

1) Characteristics of associations involved: This pertains to the extent to which the type of association (e.g., traditional healers, religious and traditional leaders, etc.) influences the success of implementation in TB control. We hypothesized that the organizational capacity is related to the characteristics of the association. We have therefore replaced the fourth domain (characteristics of individuals) by characteristics of associations involved.
2) Support system: This includes the recruitment process, training, and technical assistance that were put in place (10).

Table 1 presents the adapted constructs from the CFIR. The systematic review conducted by Kirk (2016) revealed that the CFIR can be applied at all stages of the implementation process, with particular utility in the post-implementation phase. This further justifies its use in our study (11).

**Table 1:** Domains and constructs adapted of CFIR.

Domains	Constructs
DOMAIN 1: the program's characteristics	- Origin - Qualité and strength of evidence - Relative advantage - Adaptability, complexity - Quality of design and presentation
DOMAIN 2: The outer context	- Cosmopolitanism or the level of networking of the implementing - Organization with other organizations, peer pressure and external policies and incentives.
DOMAIN 3: The inner setting	- Culture, compatibility and relative priority of the intervention goal setting and feedback structures, leadership commitment and implementation climate.
DOMAIN 4: The characteristics of the implementing actors (PR, SR, associations, health workers, NTP actors)	Beliefs, knowledge, self-efficacy and personal attributes of individuals
DOMAIN 5: The Implementation process	Stages of implementation
	- Planning, execution, reflection and evaluation, and the presence of key actors(leaders, stakeholder engagement and project champions).
DOMAIN 6: The characteristics of Civil Society Organization	Type of association, the vision, the objectives of the association
DOMAIN 7: The support system	- Training received, supervision and technical support provided.

### 2.3 Study design and setting

Our study employs a single case study design using the framework method, which provides a valuable approach to understanding the complexities of real-life situations. The case under investigation concerns the implementation of the Community TB program in the central region of Burkina Faso. This qualitative study was conducted in the capital of Burkina Faso(Ouagadougou), involving five (5) health districts. We conducted semi-structured interviews with key informants. The factors influencing the implementation were analyzed retrospectively after the end of the program.

### 2.4 Sampling and recruitment

For in-depth interviews (IDIs), we used purposive sampling to ensure a comprehensive range of perspectives and experiences regarding the implementation of the intervention. Key informants from all relevant groups were thoughtfully selected to participate in the study. The central region of Burkina Faso was chosen to ensure the inclusion of a substantial number of key informants who had been actively involved in the program.

To achieve representativeness of each group, we included all five (5) districts within the selected survey area. Systematically, we incorporated all health facilities and members of associations that had implemented the intervention in the following health facilities: Bogodogo, Boulmiougou, Kossodo, Paul VI, and Samandin.

Individuals with valuable knowledge and experience in the implementation of the community TB program were invited to participate in the interviews. The interviews were conducted until data saturation was reached, signifying that no new information or perspectives were emerging, ensuring a comprehensive exploration of the subject matter.

### 2.5 Participants

he interview participants encompassed a diverse group of stakeholders involved in the Community TB program. These included the coordinators of the association networks or associations, the presidents of the associations and the teams responsible for implementing the program, such as program managers, monitoring and evaluation managers, and financial officers. In addition, representatives from the Ministry of Health, including those in charge of health programs (NTP, PADS: health support program) and those responsible for monitoring and evaluation, were interviewed.

Health workers from all the Diagnosis and Treatment of TB Centers (DTCs) were actively enrolled in the study to capture perspectives from various healthcare settings. Furthermore, the study involved animators of associations, traditional healers, and TB patients. In this study the terms of “animators, community health workers (CHWs) were used to design them.

### 2.6 Data collection

All participants were contacted by e-mail and then by telephone. They were invited to take part in face-to-face interviews. Prior to the interviews, participants gave their consent and all interviews were recorded.

We used semi-structured interview guide tailored to each category of interviewee. These guides were created in accordance with the themes of our conceptual framework, with a primary emphasis on implementation factors. Alongside the recordings, we also took comprehensive notes during the interviews to complement the gathered data. Furthermore, we established a data extraction grid for gathering pertinent information from program documents.

The interviews were conducted until data saturation was reached. They were carried out in either French or Moore, the local language, based on the participants’ language preferences. To enhance the reliability and validity of the data, we employed a triangulation approach by also gathering information from program documents, such as activity reports and progress reports. This enabled us to cross-reference and validate the insights obtained through interviews with data from official records.

### 2.7 Ethical considerations

The study received authorization from the National Ethics Committee for Health Research of Burkina Faso under reference number 2017-4-40 on 3rd May 2017. Before conducting each interview, participants were fully informed about the purpose of the study, and their participation was entirely voluntary. They were assured that they had the right to stop the interview at any time and for any reason, without any repercussions.

Informed consent was obtained from each participant before the interviews. The interviews were digitally recorded to ensure accurate representation and were later transcribed verbatim for analysis. To protect the participants’ privacy, anonymity was strictly maintained, and no identifying references were included in the transcripts or any study-related materials.

### 2.8 Data analysis

All interviews underwent directed thematic analysis, with each interview serving as the unit of analysis. We transcribed the audio recordings of the interviews verbatim in French, and used NVivo Software version 12 for analysis.

We adopted a deductive approach for thematic analysis, creating codes and themes aligned with the dimensions of the adapted CFIR framework. Initially, we comprehensively reviewed all transcripts and took notes to gain a deep understanding of the data.

Next, we established nodes based on the seven domains of the adapted CFIR framework, including sub-nodes for the constructs. These sub-nodes were directly integrated into the previously established nodes for barriers/obstacles or facilitators. For transcript segments that didn't align with any CFIR constructs, we generated new codes. We then categorized the collected data within the CFIR domains and constructs, following the deductive approach.

Moreover, themes that couldn't be categorized within the CFIR constructs were related to the characteristics of the organizations/institutions involved and the support system in place (recruitment, training, and technical assistance). We systematically integrated this additional information into our conceptual framework.

The first author and a specialized assistant proficient in interview methodologies conducted the interviews. In tandem, a comprehensive codebook was devised in alignment with the selected conceptual framework. The coding process was executed in accordance with the guidelines stipulated in this codebook.”

## 3 Results

We conducted key informant interviews with a total of twenty-three program implementers, representing various levels, such as health center staff, and community representatives.

The results are organized in accordance with the seven dimensions outlined in our conceptual framework. The data are presented within these dimensions, which encompass the following: the program's characteristics, the outer setting, the inner setting, the characteristics of the involved actors, the Characteristics of Civil Society Organization, the implementation process and the support system.

Within each dimension, we have categorized emerging factors that could have influenced the implementation of our intervention as either barriers or facilitators. These factors offer insights into the challenges and enabling factors encountered throughout the implementation process.

### 3.1 DOMAIN 1: the program's characteristics

#### 3.1.1 Factors arising from the characteristics of the program that have facilitated its implementation

The community-based TB program was successful in its implementation due to its characteristics. This was a key domain, with three constructs emerging as important to implementation: origin, adaptability, relative advantage, design Quality & Packaging.

#### 3.1.2 Origin

The program was developed by the stakeholders involved in the fight against TB in the country. The Ministry of Health, in collaboration with its national and international partners, participated in writing the project proposal for funding request to the Global Fund (GF).

#### 3.1.3 Adaptability

During the interviews, the majority of participants highlighted the program's adaptability. Despite its complexity, involving multiple actors from various domains, the community TB program demonstrated a capacity to adjust to local conditions, thus facilitating its optimal implementation.

“*I believe the program remained flexible. At times, we made adjustments and modified the organization within associations, for example, to ensure continued implementation.”* Program Manager, SR1

However, it was evident that the adaptability of the program was a subject of debate, as actors from the Principal Recipient (PR) did not share the same opinion.

“*…we had the impression that with this program, everything was tightly structured, from the indicators to the strategies…we didn't have much flexibility…”—*Program Manager, Principal Recipient (PR).

#### 3.1.4 Relative advantage

This community-based TB program, which leveraged associations, was aligned with the objectives of the National TB Program. The primary aim was to proactively seek out TB cases within the community and improve patient management.

Overall, healthcare providers and TB patients expressed profound appreciation for the services offered by Community Health Workers (CHWs) and were highly receptive to the support they received. A significant advantage of the program, as underscored by their feedback, was the direct assistance provided by CHWs. These encompassed activities such as actively screening for suspected TB cases in the community, delivering health-related education, and providing valuable psychological support to patients dealing with stigma.

“*Prior to the engagement of associations, the district had never managed to reach 50 patients per year; however, with their* participation*, we are now able to reach 100 patients per year*”. DTC worker 3

#### 3.1.5 Design quality & packaging (perceived excellence in how the intervention is bundled, presented, and assembled)

The initiation of the project engaged a wide range of community stakeholders, encompassing traditional healers, members of civil society, political and religious figures, as well as individuals who had previously experienced TB. These diverse groups, each esteemed in their own right, played pivotal roles in supporting the project's implementation through advocacy and awareness campaigns, rendering their contributions indispensable in facilitating the entire process.

“*What worked exceptionally well was the involvement of all community components; there was a sense of synergy in our efforts…when the religious leaders spoke, their influence resonated throughout the places of worship, and it was truly remarkable.”*—Animator X

He elaborates further, stating, “*…having personally experienced TB, I can relate to the right words to convince a suspected case or a treatment-refusing patient to seek assistance at the health centers…moreover, these individuals serve as tangible proof that TB can be successfully treated when the treatment plan is diligently followed*…”

According to certain interviewees, the program's adherence to the existing healthcare system structure played a pivotal role in facilitating its implementation.

“*It was well-designed…from the peripheral level to the central level….there were established community structures to execute the work…and at each tier of the healthcare system, specific supervisors were assigned for every type of community actor.”—*PR ME Officer

### 3.2 DOMAIN 2: the outer setting

In the CFIR framework, the outer setting refers to the economic, political, and social context in which an intervention takes place, including formal and informal support systems. Our evaluation focused on networking, patient needs and resources, and external policies and incentives as defined in the CFIR framework. Networking refers to the level of familiarity between the implementers of the intervention within an organization and external entities.

#### 3.2.1 Factors in the outer setting of the community tuberculosis program that facilitated implementation

##### 3.2.1.1 Patient needs & resources

Community awareness and care activities were tailored to meet the specific needs of patients.

“*We honor the decisions made by TB suspects and patients. In many cases, we go to their homes as necessary, offering psychological support and educating their families about issues related to stigma.”*—Animator BS2

##### 3.2.1.2 Cosmopolitanism or the level of networking of the implementing

Cosmopolitanism or networking among implementers has contributed to program facilitation. Some civil society organizations have secured extra funding from external partners to address program implementation gaps.

“*We secured additional resources for the monitoring and evaluation aspect of Round 8 of GF funding, originally not part of the plan. This allowed us to provide implementing associations with computers and software*.”—Program Officer 1

##### 3.2.1.3 External policy & incentives

The external political environment strongly supported community involvement in health promotion activities. The Global Fund (GF), a major donor in the fight against HIV, TB, and malaria, motivated countries by offering dual-track funding for both the public and community sectors. Significant funding was dedicated to support community efforts against TB.

The government of Burkina Faso displayed substantial commitment by allocating 25% of GF-received funds to community activities. This commitment was evident in the engagement of associations at the district level and throughout Burkina Faso's healthcare system.

“*During Round 8, substantial resources were allocated for program implementation, and it's worth noting the commitment of political leaders. Their availability and active participation in regional review workshops were clear indicators.”—*Program Officer 3

#### 3.2.2 Factors in the outer setting of the community tuberculosis program that hindered implementation

##### 3.2.2.1 Peer pressure

Certain associations, previously involved in grant implementation, were excluded this time. Despite their voluntary community services, this exclusion created competitive pressures detrimental to the implementation process.

There was also contention over the role of PR assigned to PAMAC by some civil society leaders. Arguments arose, suggesting that this program did not originate from civil society.

“*At the national level, disputes occurred, and it was thanks to UNDP's support that PAMAC managed to assume the PR role there.”* Program Manager, BS2

##### 3.2.2.2 Patient needs & resources

The implementation of community care faced a significant obstacle due to the limited financial means of certain populations. Many patients couldn't fully access the services provided by Community Health Workers due to their extremely constrained resources. Challenges included difficulties in transportation to healthcare facilities and obtaining sufficient food for medication.

Additionally, irregular funding and the misallocation of resources (material, financial, and human) between central and peripheral levels posed vulnerabilities to the optimal implementation of the community program.

“*It's challenging; the associations lack consistent resources for transportation during home visits. The willingness is there, but resource limitations create difficulties. Sometimes, we (health workers) chip in to assist the facilitators (laughs).”*—DTC Nurse

### 3.3 DOMAIN 3: the inner setting

In the CFIR framework, the inner setting refers to the structural attributes and cultural aspects of the environment that can impact implementation (12). Key CFIR constructs relevant to the inner setting in our study included readiness for implementation, networks and communications, available resources, compatibility, relative priority of the intervention, goal setting and feedback structures, as well as leadership commitment and the implementation climate.

#### 3.3.1 Factors in the inner setting of the community tuberculosis program that facilitated implementation

##### 3.3.1.2 Readiness for implementation

Readiness for implementation encompasses leadership involvement, which includes commitment, participation, and accountability of leaders and managers, as well as the availability of resources and access to information and knowledge.

Respondents highlighted that the Principal Recipient's (PR) extensive experience in managing community programs, the competence of its staff, its institutional affiliation with the United Nations Development Program (UNDP), and its collaboration with the National TB Program (NTP) significantly facilitated the implementation process.

“*We had a PR who had previously been involved in implementing the community program during round 4. This prior experience proved to be highly beneficial,”* noted a Ministry of Health official.

##### 3.3.1.3 Access to knowledge & information

The initial training not only equipped the actors with knowledge about tuberculosis (its signs, manifestations, and means of combat) but also provided insights into health program management.

Communication within the program's internal environment was robustly established. A mechanism was put in place to enable the exchange of information in both upward and downward directions. Numerous meetings were conducted at the program's outset, and regular meetings were scheduled throughout its duration to ensure efficient communication.

“*I believe that communication was effective; informative meetings were held, and all stakeholders at the central level were kept well-informed about the program's progress,”* affirmed SR2 Program Manager.

##### 3.3.1.4 Available resources

The availability of adequate resources (human, material, and financial) facilitated the successful implementation of activities. With its experience, the Principal Recipient (PR) was well-equipped to carry out this intervention.

“*I believe that PAMAC had the experience; during round 4, PAMAC was the sole Sub-Recipient, making them the most knowledgeable and competent. There were no competitors at that level. Another important factor is that PAMAC was already accustomed to collaborating with associations in the fight against HIV and TB”.* monitoring and evaluation officer

#### 3.3.2 Factors in the inner setting of the community tuberculosis program that hindered implementation

##### 3.3.2.1 High staff turnover

The high turnover of program staff has had a negative impact on implementation. The loss of qualified human resources was a real handicap to successful implementation.

##### 3.3.2.2 Leadership of PR

The selection of PAMAC as the Principal Recipient (PR) was met with varying levels of acceptance among stakeholders. PAMAC was not seen as a typical civil society organization, but rather as an international agency. This raised concerns about the contested role PAMAC played as PR. The perception of PAMAC as an international agency might create an unsuitable working climate for the PR's leadership.

*The institutional affiliation with UNDP made it appear like a UN agency…thus, some community leaders disagreed with PAMAC as the Principal Recipient.”—*Community leaders

##### 3.3.2.3 Poor collaboration between civil society organization and health facilities

The community program also encountered challenges at the peripheral level. The data collected indicates poor collaboration between DTC health workers and animators. In certain districts, animators were perceived as “rivals,” leading to interpersonal conflicts that often disrupted activities.*”…we had difficulties with our DTC manager…he didn't want to see us in the health center…for him, anyone who didn't receive training at the health school had no place here…”—*facilitator SR1

Furthermore, in specific districts, health workers demanded cash payments from CHWs before conducting sputum analysis. They perceived the referral activities of community actors as an additional burden to their workload.

“*The situation was particularly concerning in some DTCs; it's quite perplexing. “We refer suspected cases to them, and they ask us to stop because it increases their workload,”* said one animator.

Another animator added.” *This has resulted in sputum samples lying on the ground, rotting, because they don't have time to analyze them*” Association's Leader.

The interviewees described a challenging working climate characterized by the Principal Recipient (PR) having to compensate for the shortcomings of other community actors. Moreover, a significant number of health workers held a negative perception of the associations (lack of trust, underestimation of their capacity, etc.). These factors contributed to additional challenges for the community program, in addition to the increased workload reported by healthcare providers.

“*It's true that after an awareness campaign conducted by the associations, we would see a significant increase in the number of people coming in; as soon as we noticed a large crowd, we knew that there had been sensitization somewhere*.”—DTC officer

##### 3.3.2.4 Weak institutional and organizational capacity

Not all the associations working with PAMAC had the capacity to carry out the activities. They lacked the necessary institutional and organizational systems to effectively implement the program's activities.

“*I believe that even the largest structures had to develop a capacity building plan, and even then, it was the PR team that did most of the work…but that was one of PAMAC's missions….so we didn't complain*…"PR's responsible

### 3.4 DOMAIN 4: the characteristics of the implementing actors (PR, SR, associations, health workers, NTP actors)

#### 3.4.1 Factors in the characteristics of the implementing actors (PR, SR, associations, health workers, NTP actors) that facilitated implementation

Organizations consist of individuals (implementers) who bear responsibility for and can influence the implementation of an intervention. We have examined the concepts of knowledge and beliefs, along with self-efficacy, within the context of CFIR. Knowledge and beliefs pertain to an individual's attitudes and the importance they attach to the intervention, as well as their understanding of the facts, truths, and principles associated with it (13). The majority of CHWs appear to be highly motivated by their work, recognizing its significant impact on the health of the community they serve.

Individuals, depending on the type of association (traditional practitioners, TB patients, religious and customary) to which they were affiliated, had varying levels of knowledge and beliefs about the importance of the program (knowledge and beliefs about the intervention). The implementation of such an intervention appeared to carry greater significance for actors at the central level (PR, NTP, PR) than for those at the peripheral level (implementing association, DTC agent).

“*We strongly believe in the contribution of associations to the fight against tuberculosis in Burkina Faso. The impact of their contribution has been demonstrated in other countries, and we have faith in its effectiveness here as well.*” NTP agent

“*Most associations just make noise. Furthermore, they are accustomed to seeking easy money, similar to what happened during the days of HIV*…DTC agent

#### 3.4.2 Factors in the characteristics of the implementing actors (PR, SR, associations, health workers, NTP actors) that hindered implementation

Some actors felt that the involvement of associations led to an increase in their workload. On the other hand, some actors were willing to put in extra effort to handle the influx of cases referred by the associations.

During the interviews, issues regarding the compatibility of Global Fund procedures with community life were evident. These procedures were found to be ill-suited to the functional capacities of the associations. One of the major obstacles was the collaboration with the health system. Despite being theoretically planned, in practice, the associations were not given due consideration.

“…*because civil society and public services should work together, but I had the impression that the civil part was always underestimated…the public part always underestimated the civil society part, as if the civil society should make efforts to adapt to what was done at the public level, but not the other way around, to adapt a little to what civil society does*…”—PR manager

### 3.5 DOMAIN 5: the implementation process

#### 3.5.1 Factors in the implementation process of the community tuberculosis program that facilitated implementation

The process from operational planning to implementation was participatory, with health and civil society actors collaborating closely on the community program. This inclusive approach to planning considers the concerns and inputs of all stakeholders involved.

“*It was very enlightening for me because I observed that the process was highly participatory—it brought together the various stakeholders and enabled them to synchronize their efforts and understand each other better right from the beginning”.* SR_3_ program Manager

At the operational activity level, the animators collaborated with the district health workers to plan and carry out the activities.

“*We mutually agree and validate a work plan for each quarter»* Association leader.

Furthermore, half of the interviewees expressed strong belief in their ability to achieve the objectives. The training they received before the activities began, along with their own experiences, instilled confidence in their capabilities to accomplish the set objectives.

“*It wasn't easy, but we put in the effort. We were confident in our actions, so we were determined to achieve our objectives. We had prior experience in similar activities, and on top of that, we received training. We believe we can only succeed in fulfilling our mission*.” Association Facilitator

#### 3.5.2 Factors in the implementation process of the community tuberculosis program that hindered implementation

Some interviewees expressed the view that the project did not align with a need expressed by the community. Moreover, they believed that the donor had exerted significant influence to impose its vision, which included the selection of indicators, intervention strategies, recruitment of associations, and provision of technical assistance.

“*…the primary obstacle lies in the concept of the project; based on my experience in community work, this is the main difficulty I have observed. It was not a need expressed by the population, yet the project was introduced to support them…”.* Program officer SR3

The project design did not consider some of the outcomes and lessons learned from the implementation of previous projects.

“*Interestingly, there were previous projects, like FORESA, that laid the foundation for community intervention in TB, but to my surprise, their outcomes and experiences were not taken into account in the current project design.*”*—NTP officer*

All managers reported that the significant involvement of the donor in the program's management had a negative impact on the implementation. It occasionally led to ambiguities in the desired management procedures as per the Global Fund's requirements.

*But I also believe that working with the Global Fund was not always easy; we received numerous emails and requests, asking us to justify various aspects. It was quite challenging, with frequent meetings required to respond to the emails from the Global Fund.* PR officer

Some actors felt that the involvement of associations led to an increase in their workload. On the other hand, some actors were willing to put in extra effort to handle the influx of cases referred by the associations.

During the interviews, issues regarding the compatibility of Global Fund procedures with community life were evident. These procedures were found to be ill-suited to the functional capacities of the associations. One of the major obstacles was the collaboration with the health system. Despite being theoretically planned, in practice, the associations were not given due consideration.

“*…because civil society and public services should work together, but I had the impression that the civil part was always underestimated…the public part always underestimated the civil society part, as if the civil society should make efforts to adapt to what was done at the public level, but not the other way around, to adapt a little to what civil society does…*”—PR manager.

### 3.6 DOMAIN 6: the characteristics of civil society organization

#### 3.6.1 Factors in the characteristics of civil society organization that facilitated implementation of the community tuberculosis program

The results revealed that individuals affected by the illness (infected or affected individuals, traditional healers) were more engaged in the activities without necessarily expecting funding from the program.

“*We have annual action plans, and we try to implement our activities using resource mobilization strategies. However, more often than not, we rely on our own resources from income-generating activities, membership fees, and other means.”*—Association member representing patients.

Associations made up of people affected by the disease, such as TB patient associations, show greater resilience and determination in the fight against the disease.

“*The fact of being infected or having lived with someone who is infected provides the basis for commitment; they understand better what the disease means. This situation can be motivating enough, which makes me stay committed with or without money.”*—NTP officer

The data further highlighted the strong involvement of traditional healer's associations in the implementation. This was evident from the statement of a CDT agent: “*The associations of traditional healers worked even without funding—seeing patients regain their health was enough for them. Their work was recognized several times by the highest authorities of the Ministry of Health with a medal and an incentive bonus. On World TB Day, they were always awarded*.”

#### 3.6.2 Factors in the characteristics of civil society organization that hindered implementation of the community tuberculosis program

According to the respondents, all associations were not able to address the challenges that awaited them. The existence of the association is not supported by a vision, by well-established objectives

“*we don't have any specific objectives, we are really open, if there is funding in such and such a field, we just orient our objectives and we will take the funding”.* Association leader

Our results highlight an institutional and organizational weakness in most of the associations. The lack of human resources has prevented them from having loyal and trained staff. The turnover of staff was the important barriers to good implementation.

According to one program officer, “*our inability to maintain trained staff has been our fundamental problem…what do you want we don't have the money to motivate them because of the frequent gaps in funding, so they will go in search of a better job offers”*.

### 3.7 DOMAIN 7: the support system

#### 3.7.1 Factors in the support system of civil society organization that facilitated implementation of the community tuberculosis program

The technical assistance provided by the PR's technical team and the National TB Program (NTP) has played a crucial role in enhancing the capacities of both the associations and the facilitators.

“*We were well supported by PAMAC; whenever we asked for technical support, they were there.*”—Association Facilitator Z

Our interviewees also mentioned the technical support provided by the PR and the Sub-Recipients (SRs) through a Fiduciary Management Agency (FMA) recruited by the Global Fund (GF). “*The GF came to help us secure funding to work…we learned a lot from them…with their support, our financial reports were accepted by the GF and they provided funding for us.*”*—*SR Manager

All the animators received comprehensive training and acquired the necessary knowledge to effectively carry out community TB control activities. They found the initial training to be highly valuable in enhancing their skills and enabling successful implementation of their work. Moreover, they benefited from regular supervision by health workers.

“*PAMAC, in collaboration with the NTP, provided us with training, and now we receive regular supervision from CDT officers*.”—Association Facilitator

This program provided a valuable opportunity for associations to enhance their organizational and institutional capacities. The implementation of interventions to combat tuberculosis also increased the visibility of these associations.

“…*this program has brought us numerous advantages; it's the first program that has supported and strengthened us at all levels…our relationship with the health services has significantly improved…*”—Association Leader

#### 3.7.2 Factors in the support system of civil society organization that hindered implementation of the community tuberculosis program

The system for recruiting associations and facilitators faced criticism from our respondents. They found the recruitment criteria to be inappropriate for community organizations, and many interviewees described the rules as “bureaucratic.”

“*For instance, the extensive documentation required favored larger associations similar to NGOs, even though it is widely known that they may not always be the most effective workers. Instead, they might be more focused on seeking funding than actual implementation*,” shared a member of Association Y.

An NTP officer also added, “*As a result, some genuinely capable organizations find themselves excluded because they may not meet all the specified requirements. This system is not well-suited for community organizations; it's too modernized and, to be honest, we don't quite understand it.*” Table 2 summarises the factors that have influenced the implementation of the program.

**Table 2:** Factors Influencing the Implementation of the Community Program.

Domains CFIR	Facilitating factors	Barriers/obstacles
1. The program's characteristics		
Origin	- Program was developed by the stakeholders in fight against tuberculosis of country: The Ministry of Health, in collaboration with its national and international partners	
Adaptability	The program was modified to adapt to the local context.	
Relative advantage	- Recognition of the undeniable benefits of community intervention by health professionals (reduction of multiple tasks; improvement of indicators in health facilities). - Compatibility with CDT objectives - The proximity of the intervention to the communities	
Intervention source	The project was initiated by the beneficiary country (Burkina Faso).	- The project does not address a need expressed by the population. - The project failed to consider lessons learned from comparable initiatives. - The project was driven by the donor, influencing the selection of indicators, intervention strategies, recruitment of associations, and provision of technical assistance.
Evidence des résultats	The project results were noticeable (increase in the number of people screened, decrease in the number of lost to follow-up cases). CDT Agents	
Design quality and packaging	The project setup involved groups of people due to their reputation (traditional healers, civil society actors, political and health authorities, former TB patients). The program followed the structure of the health system, involving community actors at all levels.	Involvement of a large number of associative structures.
2. The outer context		
Needs and resources	- Community awareness and care activities were tailored to meet the specific needs of patients.	
Cosmopolitanism	- Some civil society organizations have secured extra funding from external partners to address program implementation gaps.	
External policy and incentives	- The external political environment strongly supported community involvement in health promotion activities. - At the international level, the requirement to involve communities in the fight against diseases is recognized. - dual-track funding for both the public and community sectors. - Significant funding was dedicated to support community efforts against TB.	
Pression des pairs		- Certain associations, previously involved in grant implementation, were excluded this time - this exclusion created competitive pressures detrimental to the implementation process. - contention over the role of PR assigned to PAMAC by some civil society leaders. - Arguments arose, suggesting that this program did not originate from civil society
Patient Needs & Resources		- Many patients couldn't fully access the services provided by Community Health Workers due to their extremely constrained resources - Challenges included difficulties in transportation to healthcare facilities and obtaining sufficient food for medication - irregular funding and the misallocation of resources
3. The inner setting		
Readiness for Implementation	The Principal Recipient's (PR) extensive experience in managing community programs, the competence of its staff, its institutional affiliation with the United Nations Development Program (UNDP), and its collaboration with the National TB Program (NTP) significantly facilitated the implementation process.	
Access to Knowledge & Information	- The initial training equipped the actors with knowledge about tuberculosis and provided insights into health program management. - Communication within the program's internal environment was robustly established. - Numerous meetings were conducted at the program's outset, and regular meetings were scheduled throughout its duration to ensure efficient communication	
Available Resources	The availability of adequate resources (human, material, and financial) facilitated the successful implementation of activities	
High Staff Turnover		- The high turnover of program staff has had a negative impact on implementation - The loss of qualified human resources was a real handicap to successful implementation.
Leadership of PR		- PAMAC was not seen as a typical civil society organization, but rather as an international agency - the contested role PAMAC played as PR - The perception of PAMAC as an international agency might create an unsuitable working climate for the PR's leadership
Collaboration		Poor collaboration between civil society organization and health facilities
Institutional and organizational capacity		- Weak institutional and organizational capacity of some CSO
4. The Characteristics of the implementing actors (PR, SR, associations, health workers, NTP actors)		
Motivation	The majority of CHWs appear to be highly motivated by their work, recognizing its significant impact on the health of the community they serve.	
Knowledge and beliefs about the intervention	- Individuals, depending on the type of association (traditional practitioners, TB patients, religious and customary) to which they were affiliated, had varying levels of knowledge and beliefs about the importance of the program - greater significance for actors at the central level (PR, NTP, PR) than for those at the peripheral level (implementing association, DTC agent).	- Some actors felt that the involvement of associations led to an increase in their workload. On the other hand, some actors were willing to put in extra effort to handle the influx of cases referred by the associations. - The procedures of GF were found to be ill-suited to the functional capacities of the associations
5. The Implementation process		
	- The process from operational planning to implementation was participatory, with health and civil society actors collaborating closely on the community program - At the operational activity level, the animators collaborated with the district health workers to plan and carry out the activities. - half of the interviewees expressed strong belief in their ability to achieve the objectives.	- The project did not align with a need expressed by the community. - The donor had exerted significant influence to impose its vision, which included the selection of indicators, intervention strategies, recruitment of associations, and provision of technical assistance. - The project design did not consider some of the outcomes and lessons learned from the implementation of previous projects.
		- The significant involvement of the donor in the program's management had a negative impact on the implementation It occasionally led to ambiguities in the desired management procedures as per the Global Fund's requirements.-
6: The Characteristics of Civil Society Organization		
	- Associations with individuals affected by the illness (infected or affected individuals, traditional healers) were more engaged in the activities without necessarily expecting funding from the program. - Associations made up of people affected by the disease, such as TB patient associations, show greater resilience and determination in the fight against the disease. - strong involvement of traditional healer's associations in the implementation	- All associations were not able to address the challenges that awaited them. - The existence of the association is not supported by a vision, by well-established objectives - The lack of human resources has prevented them from having loyal and trained staff - The turnover of staff was the important barriers to good implementation.
7: The support system		
	- The technical assistance provided by the PR's technical team and the National TB Program (NTP) has played a crucial role in enhancing the capacities of both the associations and the facilitators. - The technical support provided by the PR and the Sub-Recipients (SRs) through a Fiduciary Management Agency (FMA) recruited by the Global Fund (GF). - All the animators received comprehensive training and acquired the necessary knowledge to effectively carry out community TB control activities. - The initial training to be highly valuable in enhancing their skills and enabling successful implementation of their work - Regular supervision by health workers. - A valuable opportunity for associations to enhance their organizational and institutional capacities.	They found the recruitment criteria to be inappropriate for community organizations, and many interviewees described the rules as “bureaucratic.”

## 4 Discussion

Our study aimed to identify the factors that facilitated or hindered the implementation of community TB program in Burkina Faso. The conceptual framework method was used to identify the obstacles and factors that facilitated the implementation of the community TB control program.

The community TB program discussed in this study exhibits various characteristics that influence its implementation, distributed across seven distinct domains. In domain 1, the results suggest that facilitating factors prevail. The origins of the program are rooted in close collaboration among stakeholders involved in tuberculosis control in the country, including the Ministry of Health and national and international partners. This cooperation from the stage of funding proposal to the Global Fund laid the foundation for a participatory approach and ongoing collaboration. In addition, the implementation process coordinated by a multidisciplinary team (PR, SR) was very useful to the implementation. This allowed for the training and supervision of animators in charge of operational activities, all of which helped to reframe and correct shortcomings in order to deliver quality services. These results suggest that the community program as designed had great potential for successful implementation.

The adaptability of the program was also highlighted in domain 1, where it is noted that despite its complexity involving diverse actors from various domains, the community TB program has demonstrated great flexibility in adapting to local conditions (9, 14–16). This flexibility greatly facilitated the optimal implementation of the program by considering the specific needs and challenges of each context (11, 12, 17). For example it seems that the possibility of flexible working hours or tasks are the indicators perceived by the actors as being associated with high management support (18). Several experiments have reported that program that allow for adaptation are more successful (19).

In terms of knowledge, beliefs and self-efficacy, the community relays had sufficient knowledge to carry out activities and were very enthusiastic about taking part in the program. They found the initial training very useful, enabling them to carry out their work successfully. All traditional healers are aware of the importance of primary health care and recognise the value of their involvement and the services they provide. It is therefore imperative to strengthen their commitment to this program. This will educate and inform them on how to help community members who are ill and when they should be referred to health facilities. The results of our study suggest that the successful implementation of this type of program is linked to the positive or negative attitudes of the stakeholders towards the program, training, self-confidence (the feeling of being able to meet the requirements of the program) and the enthusiasm of the stakeholders (20).

The external context, reveals that implementation has been facilitated by strong political and financial support from the Global Fund. The commitment of the Burkina Faso government to allocate a significant portion of the budget received from the Global Fund for community activities reflects a clear political will to involve the community in tuberculosis control.

Domain 3, centered on the internal context, emphasizes the importance of readiness for implementation. The extensive experience of the Principal Recipient (PR) in managing community programs, along with its close collaboration with the National Tuberculosis Program (NTP) and institutional anchoring with the United Nations Development Program (UNDP), has contributed to facilitating program implementation.

In domain 5, which deals with the implementation process, a participatory and collaborative approach among health and civil society actors has been a key element in achieving program objectives. Collaboration between animators and healthcare workers at the district level has allowed for effective planning and execution of activities (14).

However, despite these facilitating factors, the study highlighted challenges related to the characteristics of civil society organizations. Some associations lack qualified human resources and a well-established vision, which hampers their ability to effectively manage program activities.

In terms of the support system, it is mentioned that technical assistance provided by the PR's technical team and the National Tuberculosis Program (NTP) has played a crucial role in strengthening the capacities of associations and animators. However, some criticism is raised regarding the recruitment criteria for associations and animators perceived as bureaucratic.

One of great difficulty was the increase in workload due to the increase in attendance at the DTCs after the associations’ awareness sessions; no system of compensation for the additional workload was put in place, which led to conflicts between animators and health workers in some DTCs. Martin Muddu's study on barriers to integrating hypertension and HIV care made this same finding and confirmed that it was a barrier to program implementation (17). Terry and al (2020) study suggests a better alignment between workload and person adequacy (12, 17). The track record of most associations in the fight against HIV made it difficult for them to be adopted not only by communities because of stigma, but also by some health center staff, as they were seen as having accumulated financial resources from the multiple HIV program they ran. This could be an explanation for the financial compensation imposed by some DTCs workers. However, in other districts, instead of expecting financial compensation, an organization was set up to pay for community services (transport costs, motivation, etc.) during the unfunded periods. In this case, we have seen an increase in the level of implementation rather than a halt in community activities due to irregular funding.

In this study, the peripheral level faced several challenges. Although our program is compatible with the needs of the NTP, it would seem that the idea of the project was not perceived as an expressed need by the population according to several interviewees. This could affect the participation of the community and also the ownership of the project by the associations. In this situation, sustainability could be threatened. This suggests mixed positive or negative perceptions of the intervention among the implementing actors. Indeed, studies have shown the links between innovation uptake and needs. It is crucial for providers that the intervention meets their needs (20). The operational planning process to implementation was participatory, with health and civil society actors working together on the community program. But the actors in these structures at the peripheral level seemed to be on the fringes of the planning. This led to a problem of ownership of the program. The study by Tonny Zitti in Mali made the same observation (11). It is therefore normal that we have seen a refusal to accept community intervention in some DTCs.

Although the country was able to sign a financing agreement with the GF, financial resources were absent or very insufficient on the ground to carry out the activities, demonstrating the weakness of the structures in complying with the donor's financial management requirements. This was due to poor governance and financial management at the level of the implementing associations (12, 13). The lack of funding within the association for the implementation of activities has mainly affected the implementation of operational activities; time-bound interventions based on the availability of subsidies can erode the confidence of the community in organizations that employ them, further compromising the effectiveness and acceptability of such interventions in the long term (10). In the literature, the availability of financial resources has been shown to favor the implementation process (13, 14). However, this situation alone could not explain the difficult implementation; financial resources can better contribute to the implementation only if other conditions are met, such as the commitment of strategic actors, understanding of the project and the availability of financial (18, 21).

According to other studies (18, 19), insufficient or irregular payment has a considerable impact on the quality of outreach services. In addition, it leads to the turnover of trained facilitators, which can jeopardize implementation (21). All providers had adequate knowledge to conduct community-based TB activities. The facilitators found the initial training very useful in equipping them to carry out their work (14). But they were dissatisfied and demotivated by the irregular payment for their services. In the districts, the lack of knowledge and information on the involvement of associations in the fight against tuberculosis on the part of certain health workers (Physicians, nurses, etc.) prevented some associations from carrying out their activities successfully (13).

The PR was ready to coordinate the implementation of the community TB program, but the other structures (SR, SMEO) that were to accompany it did not have all the required skills. Hasson et al. have pointed out that the recruitment of structures is a moderating factor in the implementation of a program (22). In this sense, we propose that special attention should be paid to the inclusion of such organizations in a program.

### 4.1 Lessons learned and implications of this study

The form of community involvement used in this study has been well-received and has contributed to improved outcomes in TB control. However, its heavy reliance on external funding poses limitations on its scalability and long-term sustainability. It would be beneficial to mobilize national resources to support such community interventions. Furthermore, involving local actors (districts, health centers, implementing associations) in the entire process, from project design to implementation, is crucial for fostering ownership, which is essential for successful implementation.

The success of this program is significantly influenced by factors such as the characteristics of the involved structures, the support system, and the presence of strong leadership. Adapting the program's comprehension level to match that of the associative structures is vital for genuine ownership and meaningful engagement with the project.

### 4.2 Limitations of the study

Our study was conducted in only one out of thirteen health regions, making generalization challenging. However, the contextual analysis we performed included a wide range of participants from various sectors, including ministries of health, national and international NGOs, civil society, and all entities directly and indirectly involved in the implementation.

Another limitation was the time gap between the end of the project and the study, which may have introduced recall bias. To mitigate this, we used a diverse group of interviewers and extensively relied on program documents (reports, funding requests, etc.).

Using the CFIR for the analysis also has limitations, as certain areas might have been missed since the framework was not initially used to construct the interview guides. However, we made this choice to explore if new themes could emerge. The deductive analysis allowed us to identify and classify emerging themes, and these new categories could be used for similar community programs in the future. Additionally, as the CFIR was developed from a study in a medical setting, we sought a framework more suited to the community context.

## 5 Conclusion

In conclusion, our study conducted a comprehensive examination of the obstacles and facilitators encountered in the implementation of a community-based tuberculosis program in Burkina Faso. The results of this research shed light on several key factors that either contributed to or hindered the success implementation of program. Measures should be taken to mobilize national resources, strengthen the capacities of associations, and promote local ownership of the program. Special attention should also be given to improving financial management and resolving issues related to the recruitment and compensation of community health workers. For such community-based tuberculosis programs to succeed in Burkina Faso and in similar context it is essential to address these obstacles and facilitators.

## Data Availability

The raw data supporting the conclusions of this article will be made available by the authors, without undue reservation.
